# Development and Validation of an HPLC-UV Method for the Quantification of 4′-Hydroxydiclofenac Using Salicylic Acid: Future Applications for Measurement of In Vitro Drug–Drug Interaction in Rat Liver Microsomes

**DOI:** 10.3390/molecules27113587

**Published:** 2022-06-02

**Authors:** Hassan Salhab, James Barker

**Affiliations:** School of Life Sciences, Pharmacy and Chemistry, Kingston University, Kingston upon Thames, London KT1 2EE, UK; j.barker@kingston.ac.uk

**Keywords:** drug–drug interaction, HPLC-UV, method development, salicylic acid, validation

## Abstract

Salicylic acid is a key compound in nonsteroidal anti-inflammatory drugs that has been recently used for preventing the risk of hospitalization and death among COVID-19 patients and in preventing colorectal cancer (CRC) by suppressing two key proteins. Understanding drug–drug interaction pathways prevent the occurrence of adverse drug reactions in clinical trials. Drug–drug interactions can result in the variation of the pharmacodynamics and pharmacokinetic of the drug. Inhibition of the Cytochrome P450 enzyme activity leads to the withdrawal of the drug from the market. The aim of this paper was to develop and validate an HPLC-UV method for the quantification of 4′-hydroxydiclofenac as a CYP2C9 metabolite using salicylic acid as an inhibitor in rat liver microsomes. A CYP2C9 assay was developed and validated on the reversed phase C_18_ column (SUPELCO 25 cm × 4.6 mm × 5 µm) using a low-pressure gradient elution programming at T = 30 °C, a wavelength of 282 nm, and a flow rate of 1 mL/min. 4′-hydroxydiclofenac demonstrated a good linearity (R^2^ > 0.99), good reproducibility, low detection, and quantitation limit, and the inter and intra-day precision met the ICH guidelines (<15%). 4′-hydroxydiclofenac was stable for three days and showed an acceptable accuracy and recovery (80–120%) within the ICH guidelines in a rat liver microsome sample. This method will be beneficial for future applications of the in vitro inhibitory effect of salicylic acid on the CYP2C9 enzyme activity in rat microsomes and the in vivo administration of salicylic acid in clinical trials.

## 1. Introduction

A wide variety of clinical, physiological, and toxicological drugs are metabolized by human cytochrome P450 (CYP) enzymes [1]. Cytochrome P450 enzymes are a membrane-bound hemi-protein family that is biologically responsible for metabolizing a vast majority of hydrophobic xenobiotics into hydrophilic molecules [1]. Cytochrome P450 (CYP) enzymes are the main skeleton in clinical pharmacology and toxicology research [1].

The pharmacokinetic properties of a drug are a relatively important aspect in drug metabolism pathway identification [2]. The drug discovery and development pathways involve enzyme determination, which is responsible for the metabolism of a novel drug and the inhibition or induction of phase 1 and 2 enzymes by the new drug [2]. Inhibition occurs when the enzyme is inactivated or when the substrate does not bind to the catalytic site; thus, it can lead to a drug–drug interactions [3]. Currently, most drugs are oxidized or reduced by cytochrome P450s enzymes. Most P450s are expressed in the endoplasmic reticulum (ER) of organs such as the lungs, kidney, intestines, liver, and brain [2].

**Figure 1:**
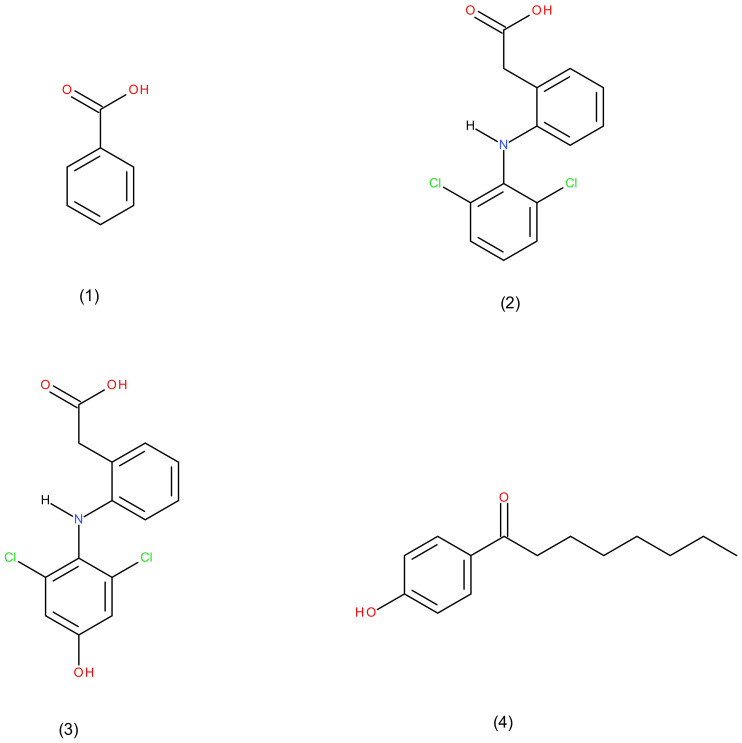
Chemical structure of (**1**) salicylic acid, (**2**) diclofenac, (**3**) 4′-hydroxydiclofenac, and (**4**) 4-hydroxyoctanophenone.

Interestingly, most marketed drugs are metabolized by six different P450 isozymes [4]. These isozymes are CYP1A2, CYP2C19, CYP2C9, CYP2D6, CYP2E1, and CYP3A4 [4]. The four CYP2C subfamilies in humans are known as CYP2C8, CYP2C18, CYP2C9, and CYP2C19 [5]. Diclofenac (Figure 1) is one of many CYP2C9 drug substrates, such as naproxen, ibuprofen, phenytoin, tolbutamide, warfarin, suprofen, and piroxicam [5]. It is known as a nonsteroidal anti-inflammatory drug (NSID) used for the treatment of rheumatoid arthritis, osteoarthritis for long term treatment, and acute musculoskeletal injury for a short period of time [6].

Cytochrome P450 (CYP2C9) is a phase 1 polymorphic enzyme that works as a major pathway in metabolizing different clinically approved drugs such as anti-bacterial, anti-inflammatory, anticancer, and antihypertensive drugs [7]. Statistically, 70% of FDA approved drugs are metabolized by the CYP2C9 enzyme [8]. An investigational study showed that four major metabolites were detected in the human urine through HPLC analysis [9]. These metabolites are 3′-hydroxydiclofenac, 4′-hydroxydiclofenac, 5′-hydroxydiclofenac, and 3′-hydroxy-4′-methoxydiclofenac [9]. A previous paper showed that diclofenac is metabolized to 4′-hydroxydiclofenac and 5′-hydroxydiclofenac in rat liver microsomes, whereas in human liver microsomes, it metabolized to only 4′-hydroxydiclofenac [10]. The metabolite 5′-hydroxydiclofenac is formed predominantly by the CYP3A4 enzyme and by CYP2C19, CYP2C8, and CYP2C18 to a lesser extent [6].

A previous investigational study showed that diosmetin (flavone) competitively inhibited the CYP2C9 enzyme activity in pooled human liver microsomes [11]. Morusin is known as a herbal plant that possesses many pharmacological effects such as anti-tumor, anti-oxidant, anti-bacterial, and anti-inflammatory properties [12]. This study showed that morusin can inhibit the CYP2C9 enzyme activity non-competitively in human liver microsomes [12]. Another in vitro inhibition study done by Li et al. (2020) [13] showed that lysionotin (natural flavonoid) does not inhibit the CYP2C9, CYP1A2, CYP2A6, CYP2E1, and CYP2D6 enzyme activities. Therefore, lysionotin was safe to be taken with other drugs that are substrates for CYP2C9, CYP1A2, CYP2A6, CYP2E1, and CYP2D6 enzymes.

Salicylic acid (Figure 1) is an anti-inflammatory drug excreted from wintergreen leaves and white willow trees that acts as an antioxidant in the pharmacological domain [14]. Aspirin is known as an antiplatelet agent that is recommended and encouraged to be taken by patients with COVID-19 [15]. A previous study demonstrated that salicylic acid and its metabolite (2,3-dihydroxybenzoic acid (2,3-DHBA), (2,4-DHBA), (2,5-DHBA), and (2,6-DHBA)) inhibited cyclin-dependent kinase (CDK 1,2,4, and 6) activity and downregulated the function of cyclin A2, B1, and D3 by targeting colon cancer tumor cells [14].

Our recent investigational studies revealed that salicylic acid has a low potential effect on CYP2C11 in rat liver microsomes [16]. In other words, salicylic acid non-competitively inhibited the CYP2C11 enzyme activity. Additionally, our previous research confirmed that salicylic acid acts as a mixed inhibitor (competitive and non-competitive) for the CYP2E1 enzyme in rat liver microsomes [17]. This means that salicylic acid has a low and high potential to cause interactions with other drugs that are a substrate of the CYP2E1 enzyme in the rat liver microsomes. According to our recent publication, salicylic acid uncompetitively inhibited the UGT2B17 enzyme activity in human supersomes [18]. This implies that salicylic acid has a negligible effect to cause a drug interaction with other UGT2B17 substrate drugs.

Recently, many studies have been reported regarding the method development and validation of diclofenac and 4′-hydroxydiclofenac in the plasma and rat serum [19]. The study by Kaphalia, et al. (2006) demonstrated a low detection limit of diclofenac and 4′-hydroxydiclofenac; however, this method was extracted by liquid–liquid extraction, which is not a simple and direct method compared with other methods for protein precipitation.

However, there are no studies regarding the method development and validation of 4′-hydroxydiclofenac with salicylic acid in the rat liver microsomes. Thus, the purpose of this paper was to develop and validate an efficient HPLC-UV method for the quantification of 4′-hydroxydiclofenac as a typical CYP2C9 metabolite using salicylic acid as an inhibitor in the rat liver microsome sample for the future evaluation of the inhibitory effect of salicylic acid on CYP2C9 enzyme activity. In this research paper, an HPLC-UV method was optimized and validated for a CYP2C9 assay using a salicylic acid inhibitor for the future investigation of the in vitro potential effect of salicylic acid on 4′-hydroxydiclofenac formation in rat microsomes.

## 2. Results and Discussion

### 2.1. Method Development

#### 2.1.1. UV–VIS (Ultraviolet-Visible) Spectroscopy

Salicylic acid (100 µM), diclofenac (200 µM), 4′-hydroxydiclofenac (100 µM), and 4-hydroxyoctanophenone (50 µM) were analyzed by UV–VIS spectrophotometry for the measurement of the maximum wavelength of each component. All standard solutions were dissolved in pure methanol (wavelength cut-off of HPLC methanol was 210 nm).

The UV–VIS spectrum below illustrates the measurements of the maximum absorbance of each CYP2C9 components.

**Figure 2:**
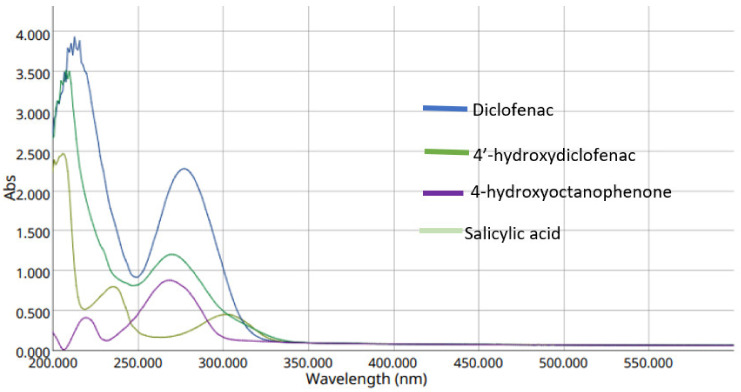
UV–VIS spectrum of salicylic acid, diclofenac, 4′-hydroxydiclofenac, and 4-hydroxyoctanophenone in the CYP2C9 assay.

According to Figure 2, the best-chosen wavelength on HPLC is 282 nm, as all of the compounds have a maximum absorption band at this wavelength. According to our previous study, salicylic acid absorbs at 282 nm wavelength (Salhab, H. et al. 2020). A study performed by Li and their colleague (2020) [13] showed that the CYP2C9 assay (diclofenac and 4′-hydroxydiclofenac) is absorbed at a 280 nm wavelength. Therefore, a 282 nm wavelength was chosen as an analytical parameter for our HPLC instrument.

#### 2.1.2. Robustness Test

A UV-HPLC method was employed to quantify the concentration of 4′-hydroxydiclofenac in rat liver microsomes. The application of this method led to higher sensitivity and lower detection limit compared with other papers in the literature. The chromatographic separation of salicylic acid, 4-hydroxyoctanophenone, diclofenac, and 4′-hydroxydiclofenac components were performed on a SUPELCO C18 column (25 cm × 4.6 mm, 5 µm particle size) using a gradient elution consisting of 0.1% formic acid in water as the mobile phase (A), acetonitrile as the mobile phase (B), and methanol as the mobile phase (C). A low-pressure isocratic elution made up of 80% methanol and 20% of water (0.1% formic acid) was used; however, the compounds were coeluted with each other. 4-hydroxyoctanophenone was chosen as an internal standard because various internal standards have been tested (caffeine and phenacetin) and the results showed that these compounds co-elute with the compound of interest when using this mobile phase. The total run time was 13 min, which was more rapid than the other method in the literature [19]. The spiked liver microsome samples were extracted by protein precipitation, which is more simple, direct, convenient, and time saving compared with the liquid–liquid extraction method [19].

##### Variation in Column Temperature

The retention time and area peak for each component were analyzed with HPLC using different sets of column temperature. Table 1 and Table 2 show the retention time and the average area peak of the components at T = 30 °C, T = 35 °C, and T = 25 °C. Based on Table 1 and Table 2, components were eluted earlier at a temperature of 30 °C. A temperature of T = 30 °C was the best-chosen analytical parameter for these compounds, as it gives a better resolution between 4′-hydroxydiclofenac and diclofenac (2.701 min) compared with T = 25 °C (2.446 min) and between 4′-hydroxydiclofenac and salicylic acid (2.691 min) compared with T = 35 °C (2.447 min). The average area peak for each component at T = 30 °C was approximately the same at T = 25 °C and T = 35 °C.

An ANOVA test with a single factor was performed to evaluate the effect of the average area peak and retention time of each component at T = 25 °C, T = 30 °C, and T = 35 °C. Based on the ANOVA test results, there was no statistically significant difference in the average area peak of each component at T = 25 °C, T = 30 °C, and T = 35 °C. The *p*-value = 0.9937 with a variance of 0.1091 at a significant level was set at α = 0.05 for T = 30 °C and the *p*-value = 0.9959 with a variance of 0.1091 was set at T = 35 °C. Therefore, changing the column temperature from T = 25 °C to T = 30 °C and T = 35 °C leaves the average area peak and retention time for each component unaffected. Thus, this method is robust for column temperature variation.

##### Variation in Flow Rate

The retention time and area peak for each component were analyzed with HPLC using different set of flow rate. According to Table 3 and Table 4, the components were eluted much earlier at 1.2 mL/min compared with 0.8 mL/min and 1 mL/min. A flow rate of 1 mL/min was the best-chosen analytical parameter for the method, as these components were eluted earlier compared with a flow rate of 0.8 mL/min. In addition, a better resolution was obtained between 4-hydroxyoctanophenone and diclofenac (0.720 min) at a flow rate of 1 mL/min compared with 0.8 mL/min (0.709 min). The resolution between 4′-hydroxy diclofenac and salicylic acid at 1 mL/min (2.691 min) was greater than the resolution between 4′-hydroxydiclofenac and salicylic acid at 1.2 mL/min (2.232 min). The average area peak for each component at a flow rate of 1 mL/min was approximately the same at 0.8 mL/min and 1.2 mL/min.

An ANOVA test with a single factor was performed to evaluate the effect of the average area peak and retention time of each component at 0.8 mL/min, 1 mL/min, and 1.2 mL/min. Based on the ANOVA test results, there was a slight statistically significant difference in the average area peak of each component at 0.8 mL/min and 1mL/min. The *p*-value = 0.7522 and the variance (0.248 > 0.05) suggest that is not statistically significant at a significant level set at α = 0.05 at 1ml/min. The *p*-value = 0.7764 has a variance of (0.224 > 0.05) at 1.2 mL/min. Therefore, changing the flow rate from 1 mL/min to 0.8 mL/min and to 1.2 mL/min keeps the average area peak and retention time for each component mostly unaffected. Thus, this method is robust for flow rate variation.

### 2.2. Method Validation

#### 2.2.1. Linearity and Range

**Figure 3:**
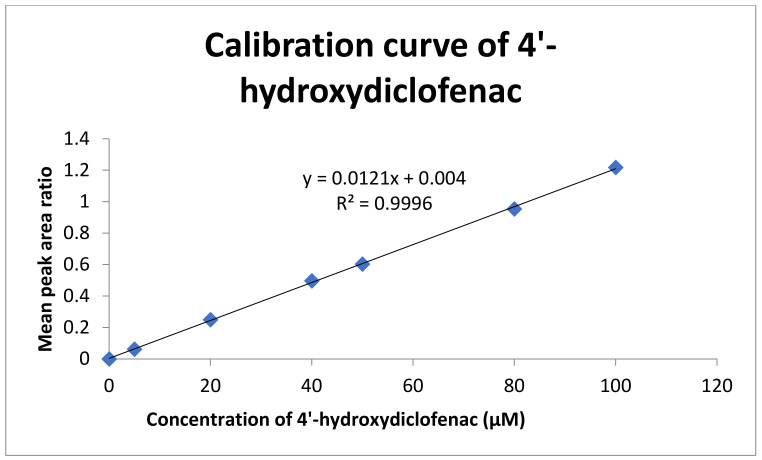
Calibration curve of 4′-hydroxydiclofenac.

A set of 4′-hydroxydiclofenac concentrations (0, 5, 20, 40, 50, 80, and 100 μM) dissolved in HPLC methanol were added in each HPLC vial containing 50 μM of 4-hydroxyoctanophenone (Internal Standard) and were injected into the HPLC instrument, and each vial was analyzed three times (n = 3). A standard calibration curve of 4′-hydroxydiclofenac was constructed using the average peak area as a function of the concentration of 4′-hydroxydiclofenac using the Microsoft Excel 2010 software system. A linear calibration curve was obtained (y = 0.0121x + 0.004, R^2^ = 0.9996) (standard error = 0.0055/intercept = 0.0064), as shown in Figure 3 below. The linear regression coefficient R^2^ > 0.99 met the analytical ICH guidelines. The linear range of 4′-hydrodiclofenac was between 5 and 100 μM.

#### 2.2.2. Limit of Detection (LOD) and Limit of Quantitation (LOQ)

The outcome summarized in Table 5 demonstrates that the CYP2C9 metabolite (4′-hydroxydiclofenac) has low detection and quantitation limit values. Moreover, the LOQ value (4.67 μM) for 4′-hydroxydiclofenac is less than the lowest concentration level of 4′-hydroxydiclofenac (5 μM). Therefore, these numerical numbers met the analytical ICH guidelines.

#### 2.2.3. Precision

##### Intra-Assay Variation of 4′-Hydroxydiclofenac in Rat Liver Microsomes

The intra-assay precision for 4′-hydroxydiclofenac was determined by quantifying three concentration levels (60, 30, and 10 μM) three times using an HPLC instrument. A calibration curve of 4′-hydroxydiclofenac at concentrations of 100, 80, 50, 40, 20, 5, and 0 μM was injected three times onto a HPLC instrument and a linear calibration curve was obtained (y = 0.0137x − 0.0003, R^2^ = 0.999) (standard error = 0.0084/intercept = 0.0084). The linear regression R^2^ > 0.99 met the ICH guidelines. The three concentration levels were determined from the calibration curve, as shown in Table 6 below. The percentage relative standard deviation was less than 5% in each concentration level according to ICH guidelines. In addition, this method showed that the protein matrix did not affect the analyte solution as it was <5%.

##### Inter-Assay Variation of 4′-Hydroxydiclofenac in Rat Liver Microsomes

Inter-assay precision for 4′-hydroxydiclofenac was determined by quantifying three concentration levels (60, 30, and 10 μM) three times on an HPLC instrument for three consecutive days (days 1, 2, and 3). A calibration curve of 4′-hydroxydiclofenac at concentrations of 100, 80, 50, 40, 20, 5, and 0 μM was injected three times using an HPLC instrument on day 1 and a linear calibration curve was obtained (y = 0.0137x − 0.0003, R^2^ = 0.999) (standard error = 0.0084/intercept = 0.0084) (see Figure S1). A calibration curve of 4′-hydroxydiclofenac at concentrations of 100, 80, 50, 40, 20, 5, and 0 μM was injected three times using an HPLC instrument on day 2 and a linear calibration curve was obtained (y = 0.0121x + 0.004, R^2^ =0.9996) (standard error = 0.0055/intercept = 0.0064) (see Figure S2). A calibration curve of 4′-hydroxydiclofenac at concentrations of 100, 80, 50, 40, 20, 5, and 0 μM was injected three times using an HPLC instrument on day 3 and a linear calibration curve was obtained (y = 0.0117x + 0.0041, R^2^ = 0.9999) (standard error = 0.0036/intercept = 0.0044) (see Figure S3).

The linear regression R^2^ > 0.99 for days 1, 2, and 3 met the ICH guidelines. The three calculated concentrations at each level were determined from the calibration curves on days 1, 2, and 3, as shown in Table 7 below. The percentage relative standard deviation was less than 10% in each concentration level according to ICH guidelines. In addition, this method showed that the protein matrix obtained ranging between 1–3% did not affect the analyte solution analysis.

#### 2.2.4. Specificity and Selectivity

A negative control blank of 50 µM of 4-hydroxyoctanophenone dissolved in HPLC acetonitrile with the spiked rat liver microsomes was injected three times to demonstrate that the method results were not affected by impurities, which determined that our method was selective.

**Figure 4:**
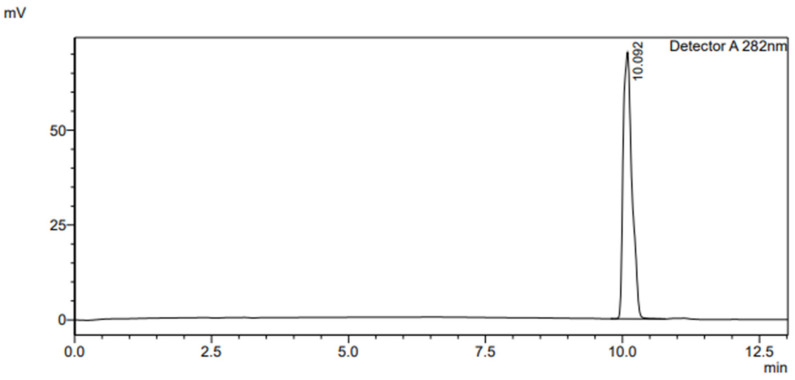
HPLC chromatogram of the negative control blank containing rat liver microsomes.

**Figure 5:**
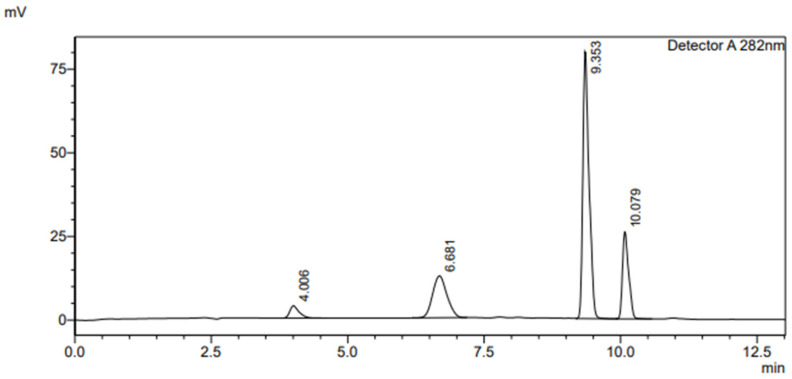
Typical HPLC chromatogram of the components at 282 nm using gradient elution programming in rat liver microsome. The peaks marked are (1) salicylic acid (100 µM) (RT = 4.006 min), (2) 4′-hydroxydiclofenac (100 µM) (RT = 6.681 min), (3) diclofenac (200 µM) (RT = 9.353 min), and (4) 4-hydroxyoctanophenone (50 µM) (RT = 10.079 min), respectively.

Achievement of specificity was done by demonstrating the best mobile phase composition (50% H_2_O in 0.1% formic acid + 15% methanol + 35% acetonitrile) using a low-pressure gradient elution system (Table 8). This resulted in a good analytical separation between the components at T = 30 °C using C18 (SUPELCO 25 cm × 4.6 mm, 5 µm) at a wavelength of 282 nm and a flow rate of 1 mL/min. Each compound containing spiked rat liver microsomes was run separately to determine the retention time of each compound at the above specified conditions (see Figure 4, Figure 5 and Figures S4–S6).

#### 2.2.5. Chromatography Fundamentals Calculations for CYP2C9 Components

It is important to calculate the chromatography fundamentals for each component for the achievement of best separation. Table 8 illustrates the calculated column efficiency (N), height plate (H), asymmetry factor (AF), and resolution (R_S_) for each CYP2C9 compound. All of the chromatography fundamentals were calculated at 100 µM for salicylic acid and 4-hydroxydiclofenac, 50 µM for 4′-hydroxyoctanophenone, and 200 µM for diclofenac.

According to Table 8, the tailing factor for each analytical compound that was obtained was less than 1.5; ICH recommendations for system suitability are set at a tailing factor of <2. Thus, these chromatography fundamentals illustrate that our methodology is suitable with a run time of 11 min. In this method, the resolution between diclofenac and 4′-hydroxyoctanophenone was 3.30 higher than the resolution between diclofenac and ibuprofen (2.06) compared with the critical value of <1.5 [20].

#### 2.2.6. Stability Test

##### Stability Test of 4′-hydroxydiclofenac in Rat Liver Microsomes

Three concentration levels (10, 30, and 60 µM) of 4′-hydroxydiclofenac were assigned as low, medium, and high levels, respectively, in the rat liver microsomes stored for 48 h under natural light conditions and were injected three times with HPLC. A range of 4′-hydroxydiclofenac concentrations (100, 80, 60, 50, 40, 30, 20, 10, and 5 µM) for each day was determined in the presence of 4-hydroxyoctanophenone (C = 50 µM). The stability outcomes are summarized in Table 9.

It is clearly shown that the calculated concentration of 4′-hydroxydiclofenac at each actual concentration level did not change significantly. Calibration curves were constructed at 0, 24, and 48 h, and they were as follows: y = 0.0115x + 0.0136 (r^2^ = 0.9994) (day 1), y = 0.0126x + 0.0099 (r^2^ = 0.9993) (day 2), and y = 0.0124x − 0.0012 (r^2^ = 0.9991) (day 3) (see Figures S7 and S8) where r^2^ met the ICH guidelines (r^2^ > 0.99). As shown in Table 9, the percentage recovery of three concentration levels at 24 and 48 h were within the acceptable recovery range (80–120%) according to ICH guidelines. In addition, the percentage accuracy of the three concentration levels of 4′-hydroxydiclofenac met the ICH guidelines (80–120%). Thus, our finding demonstrated that the 4′-hydroxydiclofenac solution was relatively stable for three consecutive days in the rat liver microsomes at room temperature, which is in agreement with the literature [21].

A previous study demonstrated that the HPLC method for the metabolism profile of diclofenac in liver slices was developed with a run time of 30 min [22]; however, the method was not optimized because of the long run time. Our method showed that diclofenac and 4′-hydroxydiclofenac using 4-hydroxyoctanophenone as an internal standard was developed and optimized with a run time of 13 min.

Various analytical techniques have been reported for the quantification of diclofenac in human plasma using the GC-MS technique [23]. The method optimized by Shah et al. (2016) showed a sensitive, robust, specific, and reproducible method for the quantification of diclofenac in human plasma. However, our method provided considerable improvement over this method, with increased accuracy and precision. Furthermore, precipitation of the protein through centrifuging without using derivatizing agents such as hexane, heptane, or benzene resulted in a high recovery >95%. In addition, the derivatizing agents used in GC-MS are quiet toxic extraction solvents [23].

A study performed on the separation of acyl glucuronide isomers of diclofenac by cyclic ion mobility spectrometry (cIM) mass spectrometry and LC-MS showed a high-throughput effective and rapid method compared with typical HPLC techniques [24]. However, the results demonstrated that 2/3 O-acyl and 4 O-acyl glucuronide isomers of diclofenac co-elute with each other, and the shape of the compound peaks is broad compared with the peak shape of the compounds.

Our HPLC method in the rat liver microsomes sample was found to be sensitive, direct, effective, low cost, and accurate for satisfying the bioanalytical method validation of ICH guidelines for the future application of pharmacokinetic in vitro and in vivo drug–drug interactions compared with other reported methods [25].

## 3. Materials and Methods

### 3.1. Chemicals

Chromatographic HPLC methanol, acetonitrile, and water were purchased from Sigma Aldrich, Co. (Old Brickyard, Gillingham, UK). HPLC analytical grade reagents 4-hydroxyoctanophenone (>98%), diclofenac (CYP2C9 substrate), and salicylic acid were procured from Sigma Aldrich, Co. (Old Brickyard, Gillingham, UK). 4′-Hydroxydiclofenac (CYP2C9 metabolite) was obtained from Carbosynth, Ltd. (Old Station Business Park, Compton, UK). Rat liver microsomes were purchased from Sigma Aldrich, Co. (Old Brickyard, Gillingham, UK) and stored at −80 °C.

### 3.2. Instruments

A UV–VIS spectrophotometry instrument with 1 cm length of quartz cuvette was used and was obtained from VWR International Ltd. (Magna Park, Lutterworth, Leicestershire, LE17 4XN, UK). The UV spectra were obtained using Chem Station Software from Agilent Technologies LDA (Cheadle Royal Business Park, Stockport, Cheshire, SK8 3GR, UK). A 570 pH meter was used and was obtained from Thermo-Fisher Scientific (Boundary Park, Hemel Hempstead, UK). An LC-2010A Shimadzu high performance liquid chromatography system (Shimadzu, Kyoto, Japan) combined with a degasser, a series 200 Peltier LC column oven, a series of 200 UV detector, a series of 200 autosampler, and an equipped quaternary gradient low-pressure pump was used for the chromatographing analysis. Target analytes were separated on a SUPELCO C18 column (25 cm × 4.6 mm, 5 µm particle size) that was purchased from Merck (Old Brickyard, Gillingham, UK).

### 3.3. Analytical Wavelength Selection

The UV–VIS spectrum was performed by preparing standard solutions of diclofenac (200 µM), 4′-hydroxydiclofenac (100 µM), and 4-hydroxyoctanophenone as an internal standard (50 µM) and salicylic acid (100 µM) in pure methanol.

### 3.4. High Performance Liquid Chromatography (HPLC) Analysis

#### 3.4.1. Rat Liver Microsomes Sample Preparation

The frozen rat liver microsomes samples were thawed at room temperature and were vortex mixed. For the specificity and robustness of the method, an aliquot of 20 µL of rat liver microsomes was vortex mixed for 2 min with 1480 µL of standard solutions containing 50 µM of 4-hydroxyoctanophenone (internal standard), 100 µM of salicylic acid, 200 µM of diclofenac, and 100 µM of 4′-hydroxydiclofenac. After centrifuging at 14,000 rpm for 10 min, 1 mL of the supernatant was transferred for the HPLC analysis.

For the analysis of 4′-hydroxydiclofenac, an aliquot of 20 µL of rat liver microsomes was vortex-mixed for 2 min with 1480 µL of standard solution 4′-hydroxydiclofenac (0, 5, 10, 15, 20, 40, 50, 60, 80, and 100 µM) containing 50 µM of 4-hydroxyoctanophenone. After centrifuging at 14,000 rpm for 10 min, 1 mL of supernatant of each 4′-hydroxydiclofenac concentration was transferred for the HPLC analysis.

#### 3.4.2. Preparation of Salicylic Acid, Diclofenac, 4-Hydroxyoctanophenone Stock and Standard Solutions in Rat Liver Microsomes

A 200 µM concentration of salicylic acid stock solution was prepared by dissolving 1.38 mg in 50 mL of HPLC-grade methanol. A 200 µM concentration of Diclofenac stock solution was prepared by dissolving it in 50 mL of methanol.

A 50 µM of 4-hydroxyoctanophenone solution was prepared by dissolving 1.1 mg powder in 100 mL of HPLC-grade acetonitrile.

#### 3.4.3. Preparation of 4′-Hydroxydiclofenac Stock and Standard Solutions in Rat Liver Microsomes

A stock of 100 µM was prepared by dissolving 1.6 mg in 50 mL of HPLC-grade methanol. A calibration curve of different concentrations (10, 15, 20, 40, 60, and 80 µM) of 4′-hydroxydiclofenac standard solutions containing rat liver microsomes were obtained. Following centrifuging at 14,000 rpm for 10 min, 1 mL of supernatant of each of the concentration levels were stored at −20 °C for the method validation.

#### 3.4.4. Liquid Chromatography (HPLC) Conditions

The chromatographic separation of salicylic acid, phenacetin, diclofenac, and 4′-hydroxydiclofenac components were performed on a SUPELCO C18 column (25 cm × 4.6 mm, 5 µm particle size) using a Shimadzu LC-2010A HT (200 UV-detector) system (Tokyo, Japan). The peak area for each component was obtained through a manual integration peak icon using the HPLC Lab Solution 2 software system. The HPLC mobile phase for separating 4-hydroxyoctanophenone (internal standard), diclofenac, 4′-hydroxydiclofenac, and salicylic acid was made up of low-pressure gradient elution system as shown in Table 10 below:

The flow rate was set at 1 mL/min using 282 nm as the wavelength for UV detection. The column temperature was set at 30 °C with an injection volume of 20 µL.

### 3.5. Data Analysis

The analytical parameters (linearity and range) for 4′-hydroxydiclofenac were determined from the calibration curves of 4′-hydroxydiclofenac using Microsoft Excel 2010 software. Calibration curve graph of 4′-hydroxydiclofenac was plotted using the average peak area ratio (average peak area of 4′-hydroxydiclofenac/average peak area of 4-hydroxyoctanophenone) as a function of the theoretical concentration of 4′-hydroxydiclofenac.

All of the experimental outcomes are represented as average ± error. All HPLC validation parameters (LOD, LOQ, % error, % accuracy, precision, % recovery, and matrix effect) were calculated using Microsoft Excel 2010 software. LOD and LOQ were calculated mathematically using the following equations: LOD = 3.3б/S and LOQ = 10б/S, where б is the standard deviation of the response and S is the slope of the calibration curve.

## 4. Conclusions

In conclusion, a new HPLC-UV method for the quantification of a metabolic product (4′-hydroxydiclofenac) was developed with salicylic acid. All of the analytical parameters for 4′-hydroxydiclofenac were validated according to ICH guidelines. In addition, this method was found to be robust. All of the analytical parameters (LOD, LOQ, accuracy, precision, % error, and recovery) for 4′-hydroxydiclofenac met the ICH guidelines. Therefore, this method (extraction by protein precipitation) is more easy, direct, time saving, and convenient than liquid–liquid extraction. This method needs to urgently be developed and validated for the future application of the in vitro potential assessment of salicylic acid on the CYP2C9 enzyme and the for the safety administration of salicylic acid with other drugs in clinical trials.

## Figures and Tables

**Table 1:** The effect of decreasing temperature on both the area peak and retention time of each component in the rat liver microsomes.

Analytical Parameters	Analytes in Sample	Average Retention Times (min)	Average Area Peak (Mean ± RSD)	Comment on Resolution
Gradient elution (50% H_2_O+ 15% MeOH+ 35% ACN), T = 30 °C	Salicylic acid (100 µM)	3.982	44,815.67 ± 0.3081	The resolution between 4′-hydroxydiclofenac and Diclofenac is 2.701 min (better resolution).
	4-hydroxyoctanophenone (50 µM)	10.094	203,645.70 ± 0.1387	
	Diclofenac (200 µM)	9.374	627,869.70 ± 0.0813	
	4′-hydroxydiclofenac (100 µM)	6.673	235,636.30 ± 0.1327	
Gradient elution (50% H_2_O+ 15% MeOH+ 35% ACN), T = 25 °C	Salicylic acid (100 µM)	4.093	45,668.67 ± 0.0599	The resolution between 4′-hydroxydiclofenac and Diclofenac is 2.446 min (good resolution).
	4-hydroxyoctanophenone (50 µM)	10.200	205,369.30 ± 0.0884	
	Diclofenac (200 µM)	9.478	633,525.00 ± 0.0322	
	4′-hydroxydiclofenac (100 µM)	7.032	233,145.30 ± 0.0952	

**Table 2:** The effect of decreasing the temperature on both the area peak and retention time of each compound in rat liver microsomes.

Analytical Parameters	Analytes in Sample	Average Retention Times (min)	Average Area Peak (Mean ± RSD)	Comment on Resolution
Gradient elution (50% H_2_O + 15% MeOH+ 35% ACN), T = 30 °C	Salicylic acid (100 µM)	3.982	44,815.67 ± 0.3081	The resolution between 4′-hydroxydiclofenac and salicylic acid is 2.691 min (better resolution).
	4-hydroxyoctanophenone (50 µM)	10.094	203,645.70 ± 0.1387	
	Diclofenac (200 µM)	9.374	627,869.70 ± 0.0813	
	4′-hydroxydiclofenac (100 µM)	6.673	235,636.30 ± 0.1327	
Gradient elution (50% H_2_O + 15% MeOH + 35% ACN), T = 35 °C	Salicylic acid (100 µM)	3.918	44,525.00 ± 0.5865	The resolution between 4′-hydroxydiclofenac and salicylic acid is 2.447 min (good resolution).
	4-hydroxyoctanophenone (50 µM)	9.975	205,037.00 ± 0.2982	
	Diclofenac (200 µM)	9.245	626,595.30 ± 0.0729	
	4′-hydroxydiclofenac (100 µM)	6.365	232,149.00 ± 0.9973	

**Table 3:** The effect of changing flow rate on both area peak, and retention time of each component in rat liver microsomes.

	Analytes in Sample	Average Retention Times (min)	Average Area Peak (Mean ± RSD)	Comment on Resolution
Gradient elution (50% H_2_O+ 15% MeOH+ 35% ACN), flow rate = 1.00 mL/min	Salicylic acid (100 µM)	3.982	44,815.67 ± 0.3081	The resolution between 4-hydroxyoctanophenone and Diclofenac is 0.720 min (better resolution).
	4-hydroxyoctanophenone (50 µM)	10.094	203,645.70 ± 0.1387	
	Diclofenac (200 µM)	9.374	627,869.70 ± 0.0813	
	4′-hydroxydiclofenac (100 µM)	6.673	235,636.30 ± 0.1327	
Gradient elution (50% H_2_O+ 15% MeOH+ 35% ACN), flow rate = 0.8 mL/min	Salicylic acid (100 µM)	5.017	53,803.50 ± 0.9971	The resolution between 4-hydroxyoctanophenone and Diclofenac is 0.709 min (good resolution).
	4-hydroxyoctanophenone (50 µM)	11.213	250,260.50 ± 0.9400	
	Diclofenac (200 µM)	10.504	779,628.00 ± 0.8101	
	4′-hydroxydiclofenac (100 µM)	8.193	289,811 ± 1.0234	

**Table 4:** The effect of changing the flow rate on both retention time and area peak of the components in rat liver microsomes.

	Analytes in Sample	Average Retention Times (min)	Average Area Peak (Mean ± RSD)	Comment on Resolution
Gradient elution (50% H_2_O+ 15% MeOH+ 35% ACN), flow rate = 1.00 mL/min	Salicylic acid (100 µM)	3.982	44,815.67 ± 0.3081	The resolution between 4′-hydroxydiclofenac and salicylic acid is 2.691 min (better resolution).
	4-hydroxyoctanophenone (50 µM)	10.094	203,645.70 ± 0.1387	
	Diclofenac (200 µM)	9.374	627,869.70 ± 0.0813	
	4′-hydroxydiclofenac (100 µM)	6.673	235,636.30 ± 0.1327	
Gradient elution (50% H_2_O+ 15% MeOH+ 35% ACN), flow rate = 1.2 mL/min	Salicylic acid (100 µM)	3.338	36,188.67 ± 0.2316	The resolution between 4′-hydroxydiclofenac and salicylic acid is 2.232 min (good resolution).
	4-hydroxyoctanophenone (50 µM)	9.256	171,764.30 ± 0.1622	
	Diclofenac (200 µM)	8.517	521,222.00 ± 0.0992	
	4′-hydroxydiclofenac (100 µM)	5.570	191,386.00 ± 1.0291	

**Table 5:** LOD and LOQ for 4′-hydroxydiclofenac in rat liver microsomes.

CYP2C9 Metabolite	4′-Hydroxydiclofenac
Limit of Detection (LOD)	1.54
Limit of Quantitation (LOQ)	4.67

**Table 6:** Intra-assay variation for 4′-hydroxydiclofenac (n = 3) in rat liver microsomes.

4′-Hydroxydiclofenac Standard (μΜ)	Calculated Concentration (μM)	Standard Deviation	% Relative Standard Deviation (RSD)	Matrix Effect (%)
Low-activity standard (C = 10 μM)	10.0949	0.0447	0.4430	0.94
Medium-activity standard (C = 30 μM)	30.8203	0.1147	0.1145	2.73
High-activity standard (C = 60 μΜ)	57.8351	0.0587	0.1016	3.61

**Table 7:** Inter-assay precision of 4′-hydroxydiclofenc (n = 3) in the rat liver microsomes.

4′-Hydroxydiclofenac Standard (μΜ)		Mean Area Peak (n = 3 Each Level)	Calculated Concentration (μM)	Standard Deviation	% Relative Standard Deviation (RSD	Matrix Effect (%)
Low-activity standard (C = 10μM)	Day 1	0.1358	10.2351	0.5348	5.2254	2.35
	Day 2	0.1369				
	Day 3	0.1185				
Medium-activity standard (C = 30 μM)	Day 1	0.4172	30.3758	1.0015	3.2970	1.25
	Day 2	0.3857				
	Day 3	0.3446				
High-activity standard (C = 60 μΜ)	Day 1	0.7854	58.7187	0.9750	1.6605	2.14
	Day 2	0.7246				
	Day 3	0.7000				

**Table 8:** Calculated analytical parameters for the CYP2C9 components.

Analytical Components	Retention Time (R_t_)	Width at Half Peak Height (W_0.5_)	Column Efficiency (N)	Asymmetry Factor (AsF)	Plate Height (H) (cm)	Resolution (R_s_)
Salicylic acid	4.00	0.18	127	1.47	0.196	6.63 7.48 3.30
4-hydroxydiclofenac	6.67	0.29	129	1.10	0.194	
Diclofenac	9.34	0.12	431	1.40	0.058	
4′-hydroxyoctanophenone	10.07	0.12	457	1.36	0.055	

N = 5.54 (R_t_/W_0.5_); H = column Length/N. AsF = B/A, where A is the distance from the leading edge of the peak to the midpoint of the peak measured at 10% of peak height, and B is the distance from the midpoint of the peak to the trailing edge of the peak measured at 10% of the peak height. Rs = 2Δt_R_/0.5(W_1_+W_2_), where W is the width at the peak base.

**Table 9:** Stability test outcomes for 4′-hydroxydiclofenac in the rat liver microsomes.

Actual Concentration of 4′-Hydroxydiclofenac (µM)		10	30	60
Calculated concentration (µM)	0 h	9.3721	30.4148	59.5072
	24 h	10.4285	33.2619	64.2936
	48 h	11.2703	33.5320	63.3625
% Recovery ^a^	24 h	111.2717	109.3609	108.0433
	48 h	120.0000	110.2489	106.4787
% Accuracy ^b^	0 h	106.2790	101.3826	100.8213
	24 h	95.7150	89.1270	92.8440
	48 h	87.2970	88.2266	94.3958

^a^ % recovery = (concentration of 4′-hydroxydiclofenac at 24 h/standard concentration of 4′-hydroxydiclofenac) × 100. ^b^ % Accuracy = 100 − ((calculated concentration − actual concentration)/actual concentration) × 100.

**Table 10:** HPLC low gradient elution programming.

Time (min)	% H_2_O (0.1% Formic Acid)	% Methanol	% Acetonitrile
0–3	50	15	35
3–4	45	27.5	27.5
4–5	35	32.5	32.5
5–7	10	45	45
7–9	5	47.5	47.5
9–10	20	40	40
10–10.5	50	15	35
10.5–13	50	15	35

## Data Availability

The data presented in this study are available in this article.

## Supplementary Materials

The following supporting information can be downloaded at: https://www.mdpi.com/article/10.3390/molecules27113587/s1. Figure S1: Calibration curve of 4′-hydroxydiclofenac for intra-assay and inter-assay Day 1. Figure S2: Calibration curve of 4′-hydroxydiclofenac for inter-assay Day 2. Figure S3: Calibration curve of 4′-hydroxydiclofenac for inter-assay Day 3. Figure S4: HPLC chromatogram of salicylic acid (100 µM). Figure S5: HPLC chromatogram of 4′-hydroxydiclofenac (100 µM), Figure S6: HPLC chromatogram of Diclofenac (200 µM). Figure S7: Calibration curve of 4′-hydroxydiclofenac stability test day 1. Figure S8: Calibration curve of 4′-hydroxydiclofenac stability test day 2. Figure S9: Calibration curve of 4′-hydroxydiclofenac stability test day 3.

## Sample Availability

Samples of the compounds are not available from the authors.
