# Supplementary material for: Development and Validation of an HPLC-UV Method for the Quantification of 4′-Hydroxydiclofenac Using Salicylic Acid: Future Applications for Measurement of In Vitro Drug–Drug Interaction in Rat Liver Microsomes
Source: Molecules. 2022 Jun 2;27(11):3587. doi: 10.3390/molecules27113587 (PMC9182407; doi:10.3390/molecules27113587)
Supplement: Supplementary file 1 [file molecules-27-03587-s001.zip › molecules-1740500-supplementary.pdf]

molecules

Supplementary Materials for CYP2C9 manuscript

Development and Validation of an HPLC-UV Method for the Quantification of 4′-Hydroxydiclofenac Using Salicylic Acid: Future Applications for Measurement of In Vitro Drug–Drug Interaction in Rat Liver Microsomes

Hassan Salhab * and James Barker

School of Life Sciences, Pharmacy and Chemistry, Kingston University, Kingston upon Thames, London KT1 2EE, UK; j.barker@kingston.ac.uk
* Correspondence: k1542808@kingston.ac.uk; Tel.: +447984974741

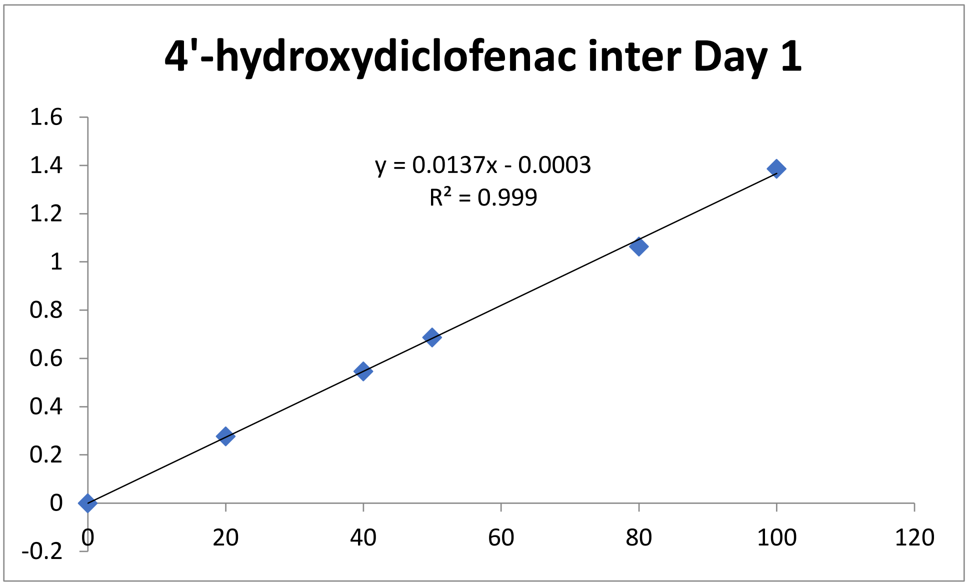

Figure S1. Calibration curve of 4'-hydroxydiclofenac for intra-assay and inter-assay Day 1.

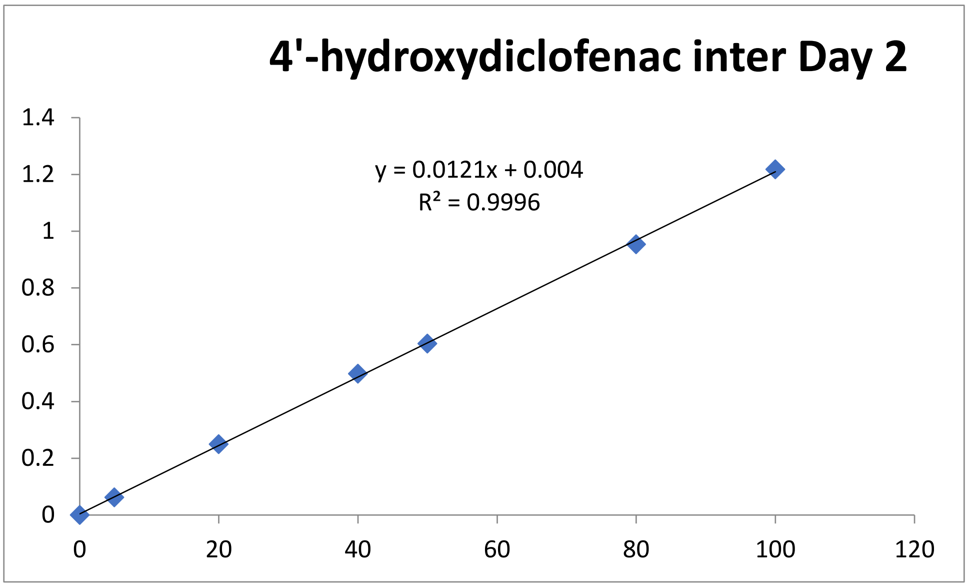

Figure S2. Calibration curve of 4'-hydroxydiclofenac for inter-assay Day 2.

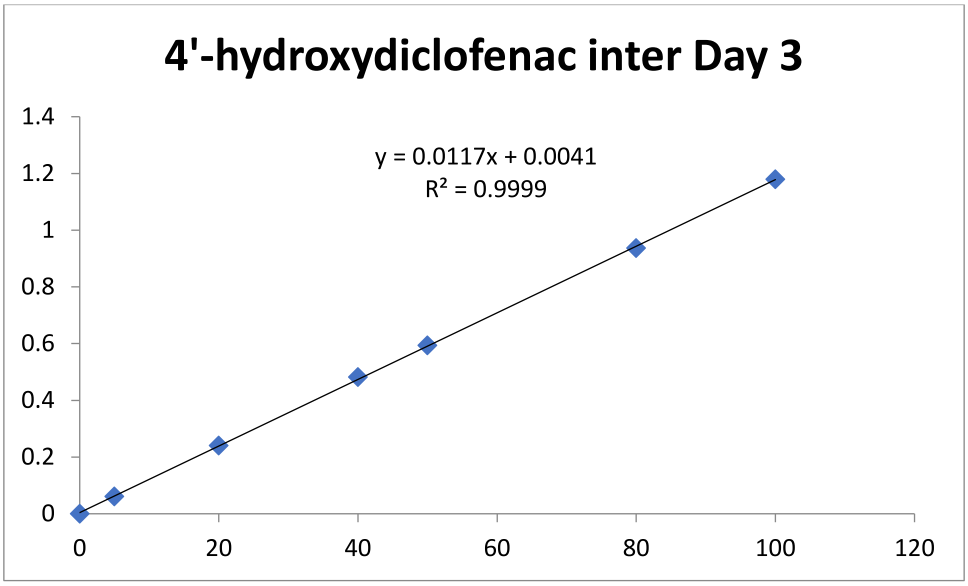

Figure S3. Calibration curve of 4'-hydroxydiclofenac for inter-assay Day 3.

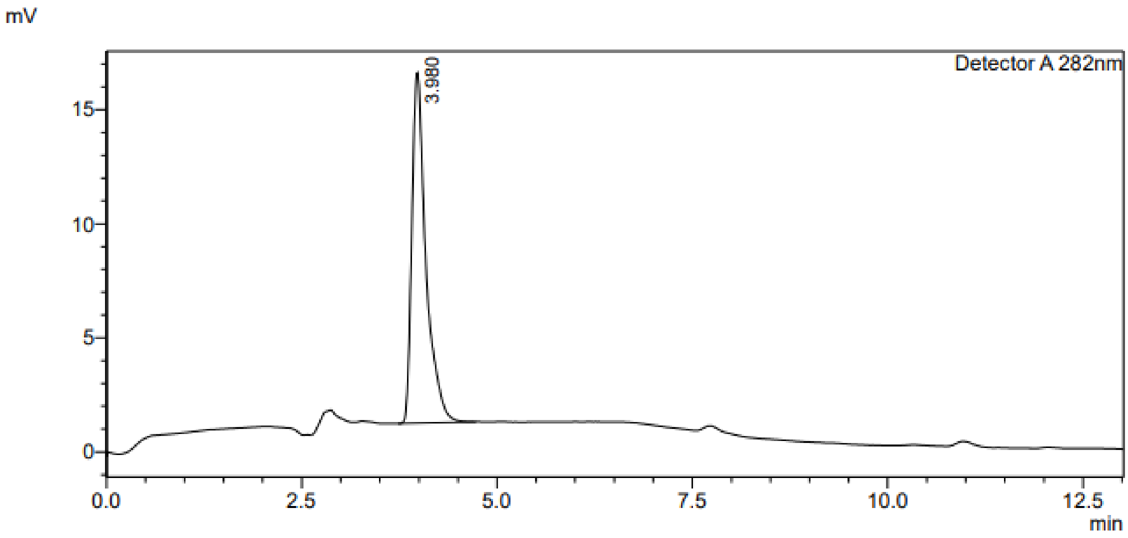

Figure S4. HPLC chromatogram of salicylic acid (100 µM).

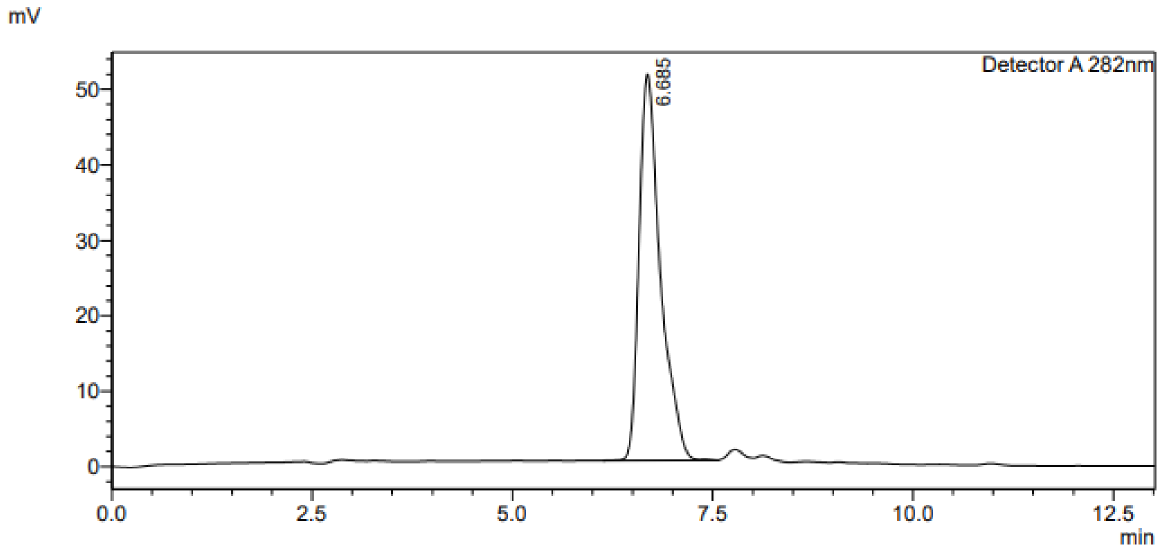

Figure S5. HPLC chromatogram of 4'-hydroxydiclofenac (100 μM).

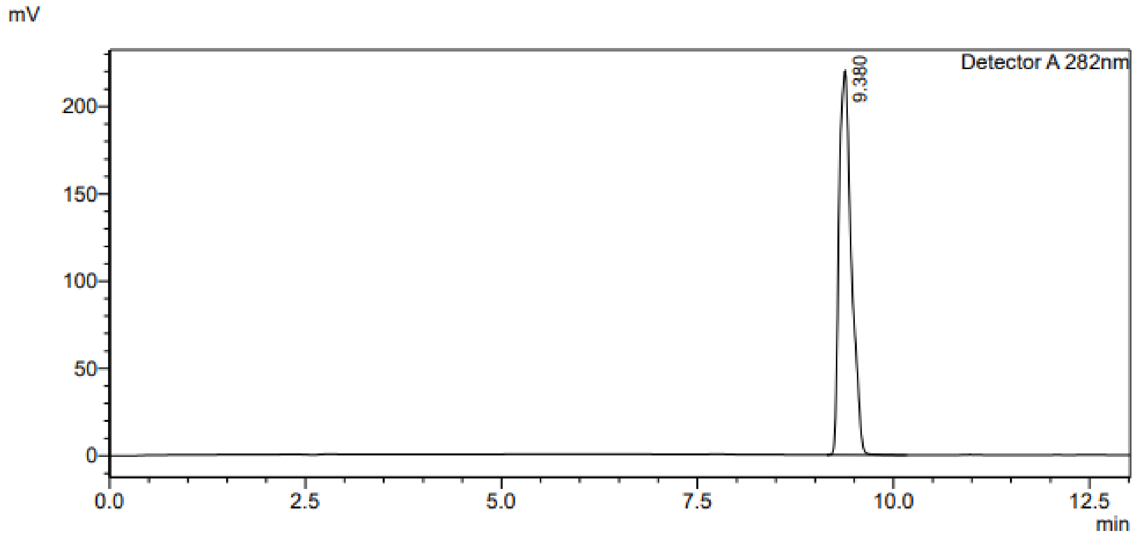

Figure S6. HPLC chromatogram of Diclofenac (200 μM).

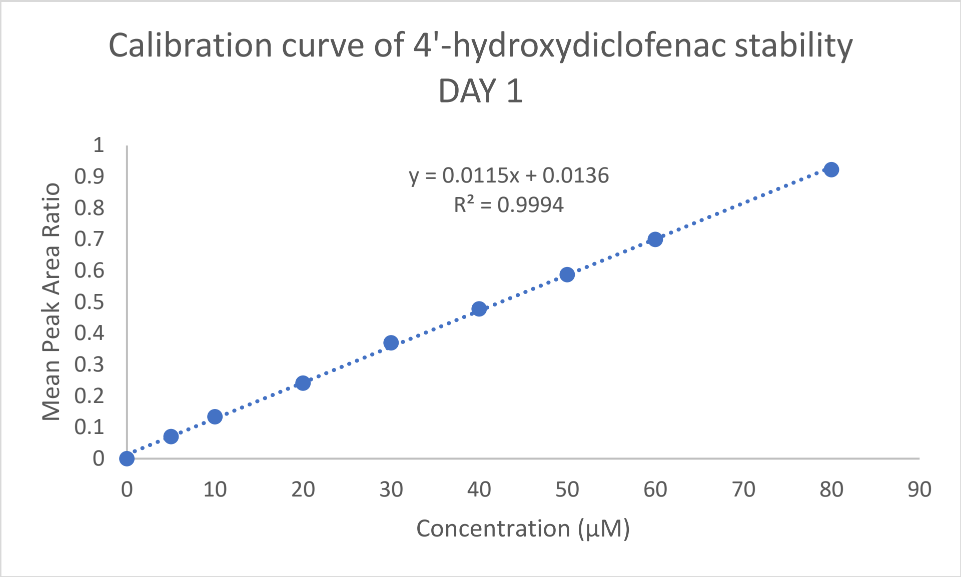

Figure S7. Calibration curve of 4'-hydroxydiclofenac stability test day 1.

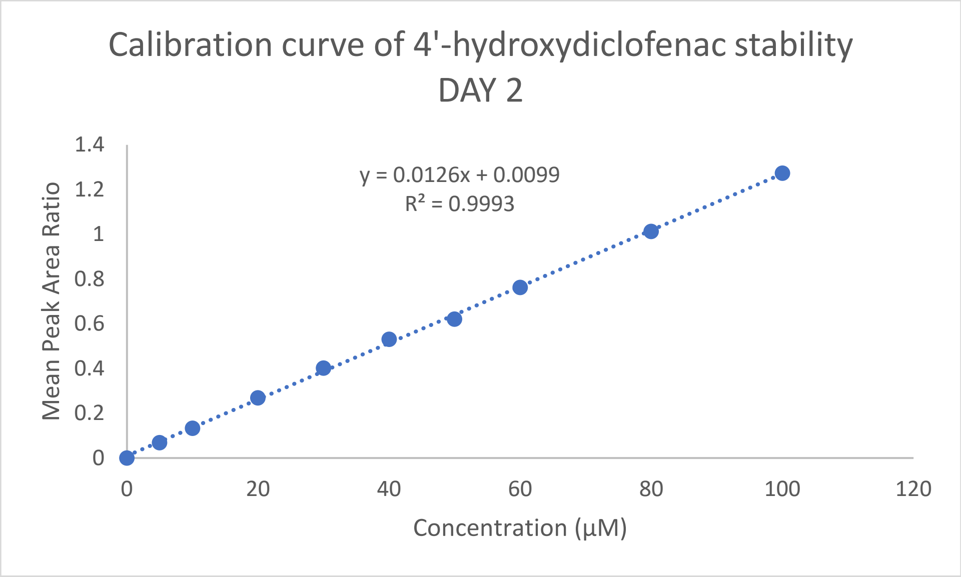

Figure S8. Calibration curve of 4'-hydroxydiclofenac stability test day 2.

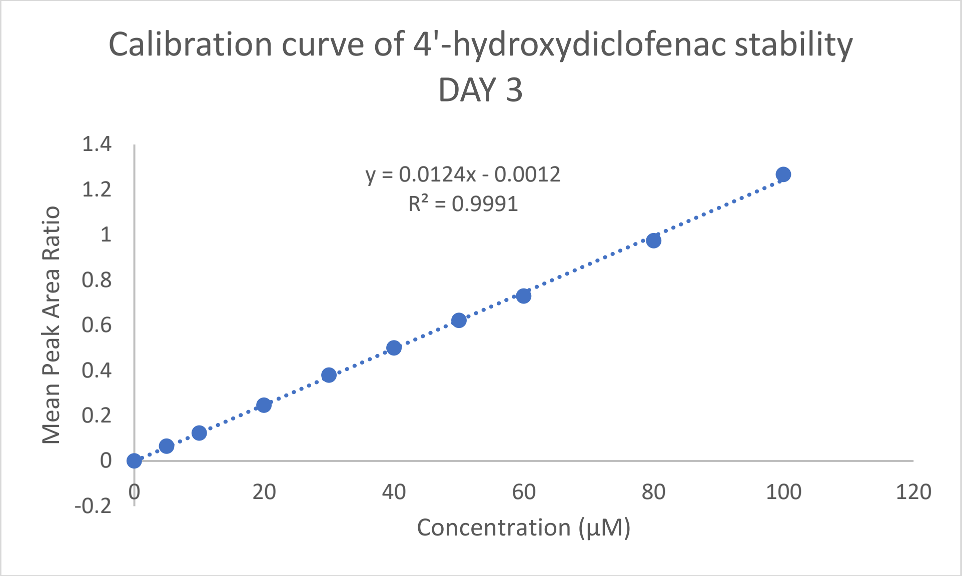

Figure S9. Calibration curve of 4'-hydroxydiclofenac stability test day 3.
